# CKD-MBD diagnosis: biochemical abnormalities

**DOI:** 10.1590/2175-8239-JBN-2021-S102

**Published:** 2021-12-03

**Authors:** Leandro Junior Lucca, Rosa Maria Affonso Moysés, Fabiana Rodrigues Hernandes, José Edvanilson Barros Gueiros

**Affiliations:** 1 Universidade de São Paulo, Faculdade de Medicina, Hospital de Clínicas, Ribeirão Preto, SP, Brasil.; 2 Universidade de São Paulo, Laboratório de Fisiopatologia, Hospital das Clínicas da Faculdade de Medicina, São Paulo, SP, Brasil.; 3 Faculdade Santa Marcelina, Hospital Santa Marcelina, Unidade de Nefrologia, São Paulo, SP, Brasil.; 4 Universidade Federal de Pernambuco, Hospital de Clínicas, Recife, PE, Brasil.

## 1. General Recommendations

1.1 The dosing methods, type of analyzed sample (blood or plasma), and sample collection and handling protocols should be considered when interpreting laboratory test results (Evidence).

1.2 Monitoring of calcium, phosphorus, alkaline phosphatase, parathyroid hormone and 25OH vitamin D [25(OH)D] in chronic kidney disease (CKD) should be started from stage 3a (Evidence). 

1.3 The dosing frequency should be established according to the CKD stage and the trend of the result, or for treatment control (Opinion).

1.4 To consider the trend, rather than an isolated value, of calcium, phosphorus, alkaline phosphatase, parathormone, and 25OH vitamin D results to guide therapeutic decisions (Opinion).

1.5 The decision for starting, adjusting or switching treatment should consider the set of biochemical parameters of mineral metabolism.

1.6 Do not consider the Ca x P product but the individual value of Ca and P to guide therapeutic decision (Evidence).

## 2. Measurement of serum calcium and phosphorus

2.1 In CKD G3a-G5D, serum calcium and phosphorus should be maintained within the normal range for the chosen method (Evidence).

2.2 Calcemia should preferably be assessed by dosing the ionized Ca (Evidence). 

2.2.1 When ionized calcium measurement is not available, calcemia could be assessed using serum albumin-adjusted total Ca (Opinion).

2.3 Serum phosphorus values up to 5.5 mg/dL are accepted for the use of active vitamin D derivatives in the treatment of hyperparathyroidism secondary to CKD.

## 3. Measurement of total alkaline phosphatase (TAP)

3.1 In CKD G3a-5, total AP measurement should be performed at least every 12 months or, more frequently, if PTH is high (Opinion).

3.2 In CKD G5D, total AP measurement should be performed every 3 months (Opinion).

3.3 In the presence of liver diseases, bone AP (BAP) measurement, instead of TAP, should be considered (Opinion). 

## 4. Measurement of parathormone (PTH)

4.1 In CKD G3a-5, optimal intact PTH levels are not yet established (Evidence). 

4.2 In CKD G5D, intact PTH levels should be maintained between 2 to 9 times the upper value of the method (Evidence).

4.3 Depending on the method of intact PTH dosing, consider the different proportions of amino-terminal (biologically active) and carboxyl-terminal (biologically inactive) fractions (Opinion).

4.4 The blood sample for intact PTH measurement should be immediately stored on ice, while serum may be stored in a freezer at -80oC for further analysis (Opinion).

## 5. Measurement of calcidiol - 25(OH)D

5.1 In CKD G3a-G5D, the serum 25(OH)D level should be in the range of 30 to 100 ng/dL (Opinion).

5.2 The blood sample for 25(OH)D measurement should be preserved under protection from light (Evidence).

## RATIONAL

It is necessary to know the methodology used by the clinical analysis laboratory, since there are different processes, materials and analysis trials, normality reference values, material transportation and packaging processes, among other factors that could interfere in the decision making regarding treatment and control of the disease^1^.

Metabolic changes in CKD-MBD become more evident from CKD G3a onward. Thus, the measurement of calcium, phosphorus, alkaline phosphatase, PTH and 25 vitamin D is recommended to be initiated at this stage and based on the frequency shown in the tables 1, 2 ^,^ 3, and 4 ^,^ ^1^. When evaluating the laboratory parameters involved in CKD-MBD, it is salutary to consider them over time, instead of the current isolated result, since the current values often represent recent attitudes. This could result in important serum changes, leading to a metabolic diagnosis disconnected from the histological pattern of the bone. The biochemical background of at least 1-2 years leads us to a trend of these variables and it is necessary for the correct diagnosis and treatment decision.

Serum calcium, phosphorus and PTH levels are influenced by several factors, including diet and dietary changes, adherence and time of drug intake, type of assay and their intra-assay coefficient of variation, circadian rhythm and the interval between the last hemodialysis session and the analysis^2^ ^-^ ^4^, in addition to collection, transport, packaging and centrifugation procedures.

Block et al^5^, in a *post hoc* analysis assessing a large cohort of patients undergoing dialysis, suggested that biochemical markers involved in CKD-MBD have limited prognostic implication, since several CKD-MBD phenotypic behaviors are observed, according to KDIGO^1^/KDOQI^6^ targets, when based on calcium, phosphorus and PTH. This highlights potential interactions among them for predicting risk of death and cardiovascular events, which reinforces the need to rely on the set of results to the detriment of the unit. Finally, it should be considered that any therapeutic attitude based on a parameter has effects, even if unintentional, on the others, as observed in the EVOLVE trial^7^. We suggest that, although there is no evidence, but based on current literature, not only calcium, phosphorus and PTH together should be considered for therapeutic decision-making, but also alkaline phosphatase.

The soluble fraction of calcium in the human body is mainly present in the extracellular fluid and distributed in the interstitial fluid and serum. The soluble fraction is found in 3 forms: a) free calcium (ionized - CaI), which is the physiologically active form and responsible for several physiological and metabolic actions; b) calcium ions bound to albumin; c) calcium bound to organic ions, such as phosphate, sulfate, and bicarbonate. Small changes in soluble calcium could result in alterations in neurological, renal, gastrointestinal, and cardiac functions^8^. Moore, however, highlights CaI as the metabolically active form responsible for biological actions in pathological and physiological conditions^3^. Thus, since the serum calcium level depends on its binding to organic anions and albumin, it is questionable whether it is preferable to consider TCa, aTCa (adjusted total calcium), or CaI as the best method to be used in clinical conditions. Formulas for calculating CaI should not be used, since they do not correlate with calcium measured directly in critically ill patients, with chronic kidney disease, hyperparathyroidism, acidemia, those receiving transfusion, and those on hemodialysis^9^ ^-^ ^13^. The formula [adjusted total calcium (aTCa) = total calcium + (4 - serum albumin) x 0.8] is suggested for correction of total calcium based on serum albumin^12^. The 2009 KDIGO^14^ suggested that serum calcium in renal function stages 3a-5D should be maintained within the normal range for the analytical method used. KDIGO 2017^1^, meanwhile, observing the evidence profile of studies that assessed serum calcium level in renal function stages 3a-5D, all observational, classified them as moderate risk of bias and low quality of evidence for clinical outcomes. Most of these studies observed elevated risk of death and cardiovascular events for increasing calcium levels, but showed no evidence for maintaining calcium levels within the normal range. Thus, the guideline only suggests avoiding hypercalcemia. Mild, asymptomatic hypocalcemia, on the other hand, could be tolerated, as it shows low risk tendency for clinical outcomes^7^. With the advent of calcimimetics, vigorous correction of hypocalcemia was initially postulated, justified by the risk of side effects secondary to it. However, KDIGO 2017^1^ highlights that the risk of replacement outweighs the risk of hypocalcemia, suggesting that it should be tolerated, provided that it is asymptomatic and without influence on the other biochemical elements involved in CKD-MBD.

Calcium concentration in the dialysate should always be individualized, considering not only serum calcium level and its need for adjustment, but also the possible histological profile of the patient. Karohl C et al.^15^ have shown that during hemodialysis, calcium balance could affect or be affected by mineral metabolism. Only two randomized controlled studies have attempted to elucidate the importance of calcium concentration in the dialysate in clinical and histological outcomes. Ok et al.^16^ submitted hemodialysis patients to a calcium concentration of 2.5 and 3.5 mEq/L for 24 months and subsequent bone biopsy. They have demonstrated a greater trend to hypercalcemia with 3.5 mEq/L calcium, a trend to reduce the coronary artery calcification score with 2.5 mEq/L calcium, and improvement in histomorphometric parameters with the use of 2.5 mEq/L calcium. Spasovsky et al.^17^ on the other hand, submitted patients with non-histologic diagnosis of adynamic disease (PTH <100 pg/mL) to calcium of 2.5 and 3.5 mEq/L in the hemodialysis bath, for 6 months, observing that those submitted to 2.5 mEq/L calcium showed better biochemical parameters, suggesting an improvement in bone mineral metabolism. However, neither has demonstrated whether an intermediate calcium concentration (3.0 mEq/L) could be beneficial in the same way. Furthermore, it is important to consider the high risk of bias in the overall, cardiovascular and cerebrovascular mortality outcomes, in addition to the low quality for evidence of results and, consequently, not demonstrating improvement in the cited outcomes.

The onset of serum phosphorus measurement during the course of CKD has been the focus of debate in literature. Although hyperphosphatemia is a late event in the CKD progression, the effect of phosphorus on PTH, calcitriol and FGF-23 is recognized. Based on observational data, phosphorus dosing during the progression of CKD and in the dialysis period is suggested, according to Table 1.

**Table 1.** Recommendation of values and serum dosing of calcium and phosphorus in CKD stages

CKD Stage	GFR (mL/min)	Serum Ca and P (mg/dL)	Dosing frequency
3a - 3b	30-59	Within reference ranges	6-12 m
4	15-29	Within reference ranges	3-6 m
5	< 15	Within reference ranges	1-3 m
5D	dialysis	Within reference ranges	1-3 m
In patients with biochemical abnormalities, the frequency of serum dosing could be increased to monitor treatment results, efficacy and side effects.			

CKD: Chronic kidney disease; GFR: Glomerular filtration rate; Ca: Calcium; P: Phosphorus

**Table 2.** Recommendation of values and serum dosing of alkaline phosphatase in CKD stages

CKD Stage	GFR (mL/min)	Serum AP (UI/L)	Dosing frequency
3a - 3b	30-59	Within reference ranges	12 m or more if PTH is high
4	15-29	Within reference ranges	
5	< 15	Within reference ranges	
5D	dialysis	1.5x upper limit of normal	3 m
In patients with biochemical abnormalities, the frequency of serum dosing could be increased to monitor treatment results, efficacy and side effects.			

CKD: Chronic kidney disease; GFR: Glomerular filtration rate, AP: alkaline phosphatase

**Table 3.** Recommendation of values and serum dosing of PTH in CKD stages

CKD Stage	GFR (mL/min)	Serum PTH (pg/mL)	Dosing frequency
3a-3b	30-59	Within reference ranges	6-12 m
4	15-29	Within reference ranges	3-6 m
5	< 15	Within reference ranges	3 m
5D	dialysis	Within reference ranges	3 m
In patients with biochemical abnormalities, the frequency of serum dosing could be increased to monitor treatment results, efficacy and side effects.			

CKD: Chronic kidney disease; GFR: Glomerular filtration rate

**Table 4.** Recommendation of values and serum dosing of vitamin D in CKD stages.

CKD Stage	GFR (mL/min)	Serum 25(OH)D (ng/dL)	Dosing frequency
3a - 3b	30-59	30-100 ng/dL	variable, according to baseline value and to monitor treatment
4	15-29		
5	< 15		
5D	dialysis		
In patients with biochemical abnormalities, the frequency of serum dosing could be increased to monitor treatment results, efficacy and side effects.			

CKD: Chronic kidney disease; GFR: Glomerular filtration rate

KDIGO 2017^1^, after reviewing the evidence from the available studies regarding the serum phosphorus level that should be adopted as a target, cites the main conclusions: (i) the association between serum phosphate and clinical result is not monotonic; (ii) there is a lack of demonstrated efficacy of phosphate binders in reducing serum phosphate in patients with CKD 3a-4; (iii) safety of phosphate binders in this population is not proven; and (iv) there is a lack of data showing that dietary phosphate restriction improves clinical results. Consequently, the work group abandoned the previous suggestion (KDIGO 2009)^14^ of maintaining phosphate in the normal range, suggesting the treatment to be focused on patients with hyperphosphatemia. The work group recognized that preventing hyperphosphatemia rather than treating it, may be useful in patients with stage 3a-5D CKD, but it acknowledges that current data are insufficient to support the safety or efficacy of such an approach and encourages research in this area.

There is still no consensus on what the "best" serum phosphorus level would be for stage 5D patients. Considering the prospective observational study COSMOS^18^, which evaluated a cohort of hemodialysis patients, the best survival was observed with serum phosphate close to 4.4 mg/dL, i.e., the upper limit considered normal in most laboratory tests. In addition, high quality of evidence has been reported linking high serum phosphorus concentrations to mortality in patients with CKD functional stage 3A-5D and after kidney transplantation^19^ ^-^ ^27^. Despite these considerations, it is accepted, for the purposes of treatment with active vitamin D derivatives, the limit of serum phosphorus up to 5.5 mg/dL. The association between high serum levels of TAP and relative risk of fractures has been reported in hemodialysis patients^28^, which justifies its monitoring, preferably in consonance with PTH. BAP correlates better with the bone formation rate, besides having a better predictive value for high and low bone turnover than PTH^29^ ^-^ ^32^. 

KDIGO 2017 recommends keeping serum PTH within normal ranges in the non-dialytic stages of CKD^1^. However, to date, there are no publications defining the best value, as well as mortality, hospitalization, and fracture outcomes. In this context, it is suggested to consider the PTH evolution over time [persistently above the upper limit of normal, or progressively increasing] in conjunction with modifiable factors (serum phosphorus, calcium and vitamin D). There is a tendency to recognize that high phosphorus intake does not always result in hyperphosphatemia, especially in the non-dialytic stages of CKD, but it may worsen SHPT.

The control of PTH elevation is necessary due to its association with morbidity and mortality and it should first go through the control of phosphorus intake (observing the phosphorus/protein intake ratio), correction of hypocalcemia, correction of hyperphosphatemia with binders and vitamin D replacement^1^ ^,^ ^33^. If PTH is not controlled with the above measures, the use of calcitriol or vitamin D analogues (paricalcitol or alfacalcidol) may be considered, especially in CKD stages 4-5. The risk of hypercalcemia^1^ ^,^ ^34^ ^,^ ^35^ and possible CKD progression^1^ are limiting factors for routine use, since the available publications show no benefits in mortality and hospitalization hard outcomes, but only in biochemical and histological control^11^ ^,^ ^35^ ^,^ ^36^.

There is no consensus on the appropriate or toxic serum level of 25(OH)D. However, the Brazilian Society of Clinical Pathology and the American Society of Endocrinology are unanimous in stating that in CKD, regardless of stage, it should be above 30 ng/mL and lower than 100 ng/mL.

The decision whether to measure, when and how often to measure, and the serum level required should be individualized according to the other CKD-MBD biomarkers condition. There is a demonstrated association between 25(OH)D deficiency (< 10 or 15 ng/mL) with several diseases^37^ ^,^ ^38^ and, in CKD, with mortality^39^. However, there is no publication showing that calcidiol repletion at a certain level reduces it. Since KDIGO 2009^14^, only one randomized controlled study has been reported, by Oksa et al.^40^, assessing cholecalciferol supplementation at high (80,000 IU/month) and low (20,000 IU/month) doses in adult patients with CKD stage 2-4, showing increased serum levels in both groups, greater in the high dose group. However, PTH did not significantly differ between them.

Routine serum calcitriol dosing is not recommended, since the analysis trials are not completely standardized, half-life is short, measurements could be artificially altered by the provision of exogenous calcitriol and vitamin D analogues, and there are no data indicating that its measurement is helpful in therapeutic definition or predicts clinical outcomes^14^.

Finally, there is not sufficient evidence to date to justify the routine serum dosing of other biomarkers, such as sclerostin, FGF-23, klotho, CTX, P1NP, in the management of CKD-MBD. 
